# Prions—Not Your Immunologist’s Pathogen

**DOI:** 10.1371/journal.ppat.1004624

**Published:** 2015-02-19

**Authors:** Mark D. Zabel, Anne C. Avery

**Affiliations:** Prion Research Center, Department of Microbiology, Immunology and Pathology, College of Veterinary Medicine and Biomedical Sciences, Colorado State University, Fort Collins, Colorado, United States of America; Washington University School of Medicine, UNITED STATES

A colleague and fellow immunologist, we will call her “Anne,” lifts her index and middle fingers on each hand and bows them in “air quotes” as she says prion “immunology” during my student’s thesis committee meeting. Anne says she works on “malaria, a real pathogen that elicits a real immune response.” Now, I am pretty sure Anne believes prions exist, but does she have a point about the immune response they elicit? The answer may surprise you.

Prions are remarkable, enigmatic pathogens that are quite different than most disease-causing entities. According to the prion hypothesis, prions are infectious agents devoid of instructional nucleic acid [1]. They propagate themselves without a genetic code, instead enciphering their infectious nature structurally, within the protein conformation itself. Mounting evidence supports the prion hypothesis, including the generation of infectious prions from purified recombinant protein [2]. Soon after Prusiner coined the term “prion,” his and Charles Weissmann’s labs discovered that a cellular gene encodes the prion agent [3]. Strangely, though, Prusiner had already demonstrated that infectious prions did not include nucleic acid, suggesting that prions infect without transmitting the gene encoding them. So attention turned to the host, in which this gene also encodes a normal form of the agent, called cellular prion protein (PrP^C^), that was later shown to be absolutely required to generate both genetic and acquired prion diseases [4]. And so, all the armchair immunologists reading this article right now pause and say, “Wait a minute…” while Anne chimes in with “prion immunology.” Here we go.

## Current Evidence for an Immune Response to Prions

Natural prion exposure most likely involves the oronasal cavity and gastrointestinal tracts, both of which rely heavily on the immune system for protection from pathogens. Prion immunologists (no snickering, Anne) have put forth tremendous effort to characterize the host—prion interaction during infectious prion disease. Strong evidence demonstrates a significant role of innate immunity in both combatting and abetting peripheral prion pathogenesis [5]. Specialized epithelial cells of the mucosal immune system called microfold (M) cells sample luminal contents and pass them to innate immune cells residing on the other side in the lamina propria. M cells can transport prions from their apical surface contacting the lumen to their basolateral side to antigen presenting cells (APCs) waiting in the lamina propria.

APCs of the mononuclear phagocyte lineage, including macrophages, monocytes, and dendritic cells (DCs), process and present the majority of antigens introduced to the host immune system. All of these cells trap prions, with macrophages comprising most of them at the inoculation site [6]. These hungry cells remain there, gobbling up and sequestering, if not fully digesting, prions and preventing them from traveling to draining lymph nodes [7]. Those prions that escape macrophagocytosis can drain into and flow through lymph fluid (the immune system’s superhighway), unattached to cells, where they are trapped by another set of macrophages minding entry into the lymph node at the subcapsullary sinus. DCs and monocytes can also escort prions into draining lymph nodes later via innate immune molecules of the Complement system, including C1q, C3, and C4. DCs and monocytes express receptors for these Complement proteins that bind prion-Complement complexes. Toll-like receptors (TLRs), another class of innate immune receptors, may also aid this process [8]. B cells express Complement receptors CD21/35 that may bind prions directly without C3, which is required to mediate CD21/35 binding to conventional bacterial pathogens [9]. Even mast cells, the histamine cannons that mediate allergic immune responses, have been proposed to facilitate prion infection because they express significant PrP^C^ and can be activated to release intracellular contents by binding C3 [5].

What happens next remains a mystery, but we do know that B cells, then another special antigen-presenting cell permanently residing in lymph nodes called follicular dendritic cells (FDCs), trap prions. B cells likely traffic prions to FDCs, which replicate them in nascent germinal centers forming in lymphoid follicles [8]. FDCs express loads of PrP^C^ and moving FDCs closer to peripheral nerves increases the speed of prion neuroinvasion [10], while deleting them largely prevents disease [11]. Several studies have also reported changes in mRNA and protein levels of cytokines and chemokines, messengers and directors of immune responses, during prion infection.

## Why No Adaptive Immune Response to Prions?

Anne rolls her eyes and says, “Yes, but what about a real immune response, one that adapts and responds to an infection with specificity and memory?” Well, that probably just does not happen. No humoral immune response to prions has been detected since researchers began looking in the early 1970s [12]. Presumably, negative selection eliminates B and T cells that recognize prions because they autoreact against PrP^C^, a self protein that shares identical primary amino acid sequence with PrP^Sc^, the misfolded, prion form [13]. While this explanation comforts supporters of the prion hypothesis, immunologists like Anne remain skeptical. One might expect an adaptive immune response against prions, since one of their defining characteristics is misfolding the normal PrP^C^ into PrP^Sc^. This misfolding, or re-folding, should create secondary and tertiary structures that could be recognized by antibodies as novel, discontinuous epitopes that would be seen as foreign. A big problem, though, lies in another defining feature of prions—their resistance to protease digestion.

## Explaining the Perverse and Often Baffling Lack of Prion-Specific Immunity

**Fig 1:**
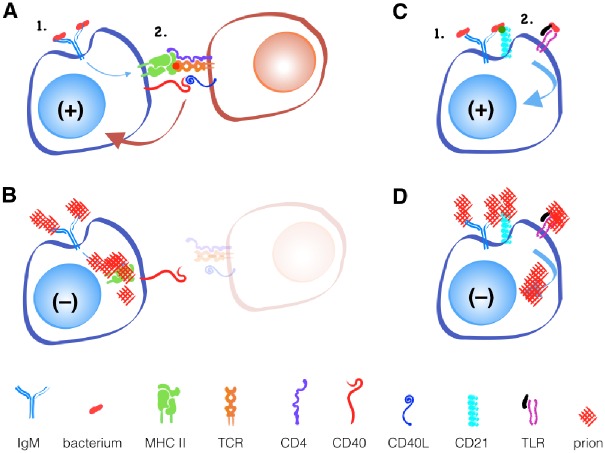
Prions fail to activate B cells with or without T cell help. A simplified model of T cell-dependent and independent B cell activation. Binding of antigen to IgM on the surface of B cells provides the first activation signal. (A) T cells that recognize antigen processed by the B cell and presented in MHC II molecules provide the second signal, typically through its CD40 ligand binding CD40 on the B cell during infections with classic pathogens. (B) Prion-specific T cells are absent during prion infection. Other innate immune receptors, like CD21 or TLRs, can provide secondary signals without T cells in response to (C) bacteria, but not to (D) prions.

The binding of a protein to an antibody on the surface of a B cell is the first step toward the development of an antibody-producing plasma cell (Fig 1). B cells respond to this activation signal by endocytosing and degrading the protein into linear peptides. Class II MHC proteins display these peptides on the surface of B cells to T cells with a receptor specific for the peptide/MHC II complex. This interaction facilitates further signals including CD40 ligand binding to CD40 to help activate B cells to differentiate into plasma cells. Thus, optimal antibody production requires that peptide antigen be processed and presented on class II MHC, and T cells exist that recognize this peptide.

While B cell receptors may recognize novel discontinuous epitopes generated by PrP^C^ misfolding to PrP^Sc^, T cells recognize only linear epitopes. Since the primary amino acid sequence of PrP^Sc^ and PrP^c^ is identical, T cells that recognize linear PrP^Sc^ will be recognized as self-reactive and will either be deleted in the thymus or controlled in the periphery via active inhibition by regulatory T cells or anergy via lack of inflammatory or other secondary stimuli.

An alternative, T cell-independent B cell activation can occur with polymeric antigens, typically non-protein antigens like bacterial polysaccharides and nucleic acids. These antigens can be recognized by a surface-bound antibody like IgM and other B cell receptors like CD21/35 and TLRs. Both receptors can provide secondary signals that help activate B cells without T cell help. If CD21/35 and TLRs can recognize prions, Anne argues, they should provide the secondary signal for B cell activation and antibody production that T cells cannot. So why does this not happen?

**Fig 2:**
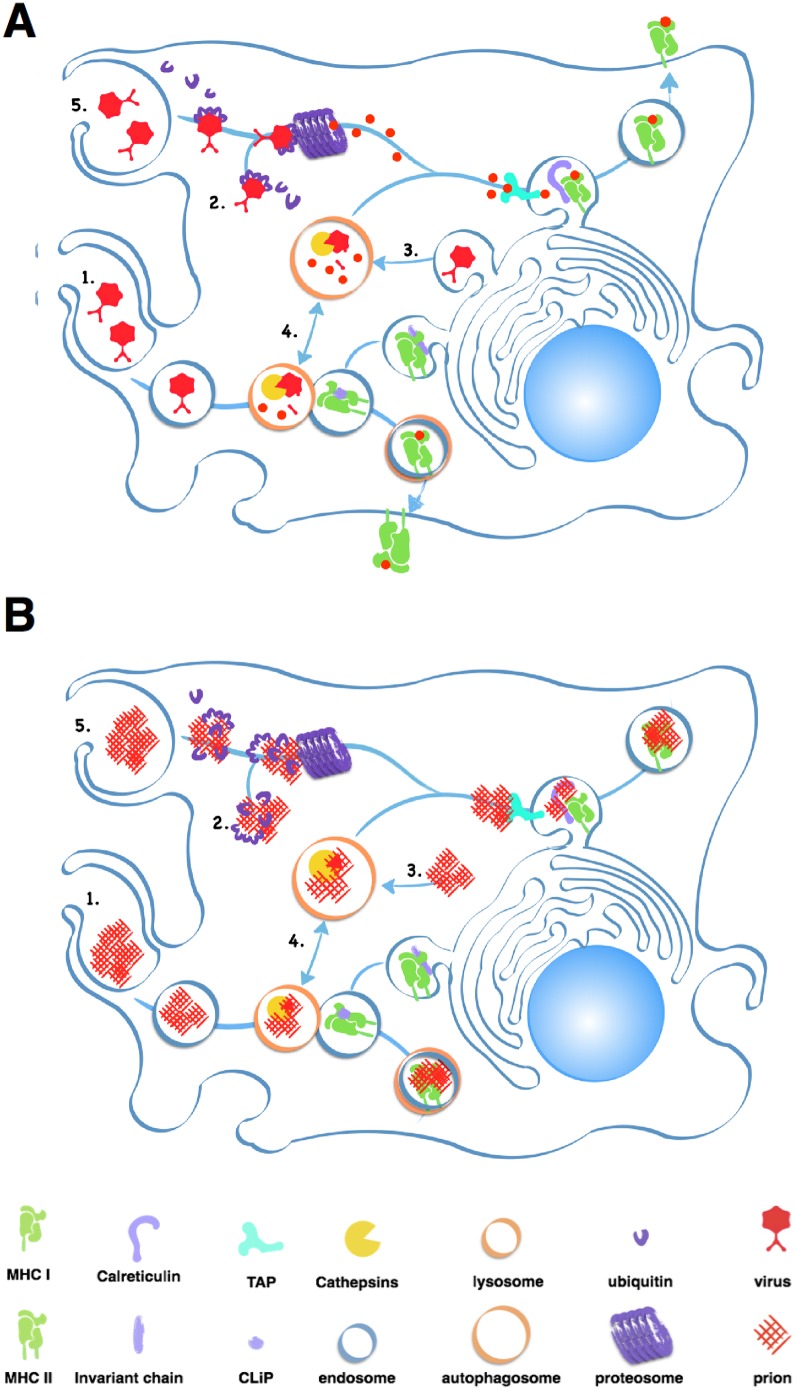
Antigen processing and presentation of classic pathogens and prions. A simplified model showing a macrophage processing and presenting viral (A) and prion (B) antigens. Each panel depicts typical antigen processing of (1) exogenous pathogens via endocytosis and fusion with acidified lysosomes, which degrade proteins into small peptides. Endolysosomes then fuse to vesicles containing MHC II molecules, which load peptides into their antigen binding clefts. Vesicles containing loaded MHC II molecules traffic to and fuse with the plasma membrane, depositing the loaded MHC II molecule on the cell surface. (2) Ubiquitin chains mark endogenous pathogens for degradation by the proteosome, which process proteins into peptides. Multiple proteins, including TAP and Calreticulin, help load peptides onto MHC I molecules and transport them to the cell surface. (3) Autophagy can degrade cellular proteins derived from the host and pathogens into peptides that can be presented by MHC I molecules. Cross presentation of (4) endogenous pathogens by MHC II and (5) exogenous pathogens by MHC I molecules via processing through the autophagosome and the proteosome occur less frequently. Prions likely constipate antigen processing at multiple points in these pathways, preventing presentation of prion-specific antigens.

APCs, including B cells, typically process large extracellular antigens like aggregated prions via the external endosomal pathway (Fig 2). Endosomes internalize antigen then fuse with lysosomes containing acidic proteases like Cathepsins and aspargine endopeptidases that digest antigens into peptides that MHC II proteins present on the cell surface. Unfortunately, while prion infected cells increase expression of Cathepsins [14], these proteases may not effectively digest prions [15,16]. Accumulating prions may impair APCs’ ability to efficiently degrade cellular components in a process called autophagy [17], further limiting antigen processing and other cellular processes that keep APCs alive and kicking.

Prion-derived peptides may also have trouble gaining access to the class I presentation pathway. Cross-presentation of exogenous antigens like prions by class I MHC proteins can occur through the proteosome, a large protein-degrading complex that degrades cytosolic antigens and self protein tagged for disposal by chains of a small protein called ubiquitin. This endogenous pathway processes both self and foreign peptides for presentation on MHC class I molecules expressed on all nucleated cells. The proteosome typically processes smaller, soluble antigens, and prions inactivate this protein digester by directly inhibiting its proteolytic domain [18].

APCs may encounter further problems presenting prion peptides on MHC I proteins. Calreticulin, a protein important for loading peptide antigens into MHC I molecules, also binds C1q, a Complement protein that binds prions [19], which would likely block antigen loading. C3 can opsonize prions and APCs can phagocytose C3-opsonized immune complexes, but without antibodies, C3-coated prions enter endosomes that do not efficiently fuse with lysosomes, preventing further processing [20].

All told, prions present unique problems for both antibody production and CD8 T cell generation. B cells that recognize novel prion epitopes created by protein misfolding fail to receive sufficient secondary stimulation to differentiate into antibody-producing plasma cells. Prions constipate and stress APCs, causing lack of antigen processing and presentation and secondary signals that may explain why even prion protein deficient mice do not elicit antibody or cellular response to the misfolded protein.

## Breaking Tolerance—Creating a Prion Vaccine

OK, so it seems that the adaptive immune system may not be able to process and/or present novel prion antigens. So what if we provide a pre-processed prion antigen, something that mimics a prion-specific epitope that APCs can present? Some success has been reported using aggregated or fragments of recombinant PrP [8,21]. Salmonella-expressing PrP stimulating mucosal immunity has been most successful so far [22]. Perhaps expressing a small, fibrillized prion peptide; prionoid amyloid; or other fibril mimetic would improve vaccine responses. Novel adjuvants like TLR agonists that provide powerful secondary signals important for robust vaccine responses, linking a prion peptide to a carrier protein like keyhole limpet hemocyanin that Cathepsins can easily cleave, or inflammatory monocyte migration inhibitors might further potentiate vaccine efficacy. Using soluble CD40 ligand as an adjuvant to bypass the requirement for T cell help may potentiate both humoral and cellular immune responses.

## Be Careful What You Wish For—The Perverse and Often Baffling Immune Response to Prions

The holy grail of an effective vaccine is sterilizing immunity mediated by powerful neutralizing antibodies. Proof-of-principle has been shown in studies promoting mucosal immunity against prions [22]. The Complement system opsonizes most of the resulting antibody-antigen complexes, marking them for disposal mainly by Kupfer cells, the macrophages of the liver. However, compelling evidence shows that Complement facilitates prion transport to germinal centers within FDCs, where efficient prion replication occurs [9,23,24]. Generating prion-specific antibodies could therefore facilitate Complement trapping and transport of prions to draining lymph nodes—the very place they replicate most efficiently.

So maybe a cell-mediated response is better. Perhaps a more effective prion vaccine stimulates prion-specific T cells in the periphery to produce inflammatory cytokines like IFNγ, that can activate macrophages to phagocytose prions and degrade or at least sequester them, as has been shown to occur [6,7]. Such an atypical, cell-induced innate immune vaccine may be just what a host needs to respond to such an atypical pathogen. Or not, says Anne.
